# Evaluating Complications of Chronic Sinusitis

**DOI:** 10.1155/2017/8743828

**Published:** 2017-01-09

**Authors:** Phillip Hong, Charles A. Pereyra, Uta Guo, Adam Breslin, Laura Melville

**Affiliations:** ^1^Department of Emergency Medicine, New York Methodist Hospital, Brooklyn, NY, USA; ^2^School of Medicine, St. George's University, West Indies, Grenada

## Abstract

Chronic sinusitis is a relatively common diagnosis throughout the US. In patients with an otherwise unremarkable medical history the treatment is typically supportive, requiring only clinical evaluation. We present the case of a 25-year-old male with a history of chronic sinusitis that was brought to our emergency department with new-onset seizure. Three days before he had presented to his usual care facility with two days of headache and fever and was discharged stating headache, subjective fever, and neck stiffness. After further investigation he was diagnosed with a mixed anaerobic epidural abscess. The evaluation and management of chronic sinusitis are based on the presence of symptoms concerning for complication. Prompt investigation of complicated sinusitis is essential in preventing debilitating and fatal sequelae. Our case study underscores the importance of early diagnosis and appropriate management.

## 1. Introduction

Chronic sinusitis affects approximately 14.6 percent of the US population and is currently the 5th most common condition treated with antibiotics, accounting for 18–22 million physician visits and costing 3.4–5 billion annually [1]. Acute sinusitis is usually viral, whereas chronic sinusitis is mostly caused by anaerobes, gram-negative bacteria,* Staphylococcus aureus*, and fungi. Both acute and chronic sinusitis require the presence of at least 2 of the following symptoms: purulent discharge from nose or nasopharynx, nasal congestion, pain, tenderness and swelling around the maxillary, ethmoid, or frontal sinuses [2]. Complicated sinusitis is suspected in patients with symptoms lasting greater than ten days, fever greater than 101°F, or extrasinus/systemic symptoms [3]. This case highlights the importance of considering intracranial abscess in the presence of red flags (fever, toxic appearance, and systemic symptoms) to prevent possible debilitating or fatal sequelae.

## 2. Case Presentation

The patient is a 25-year-old male with a past medical history of chronic sinusitis who was brought to our emergency department by EMS for new-onset seizures. The patient had been complaining of weakness, frontal headache, sinus pain, and nasal congestion for the last 5 days. He was seen at a different emergency department three days before and discharged with a diagnosis of viral syndrome. On presentation to our department the patient was postictal and complained of neck stiffness, headache, congestion, and fever; he was placed in isolation due to concern for meningitis. Initial vital signs included a temperature of 103°F, heart rate of 112 beats per minute, 20 breaths per minute, and blood pressure of 116/61 mmHg. On examination he was diaphoretic and lethargic but was fully oriented and able to answer questions. Cranial nerves two to twelve were intact with no focal neurologic deficits; the remainder of the physical examination was unremarkable except for bilateral frontal sinus tenderness to palpation.

**Figure 1:**
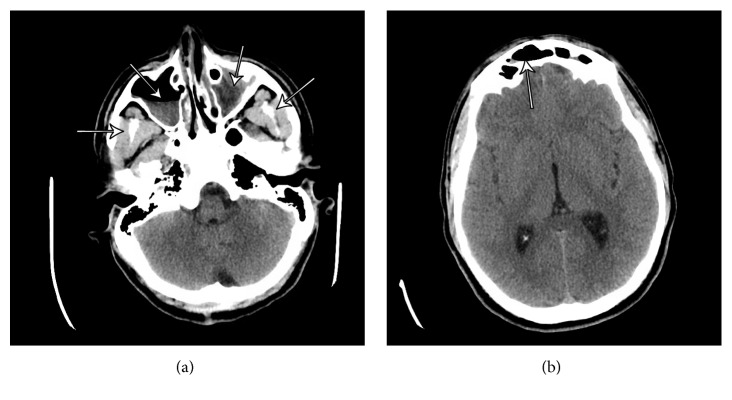
(a) illustrates severe sinusitis involving the ethmoid air cells and left maxillary sinus, moderate involvement of the right maxillary sinus, and mild involvement of the frontal sinuses. (b) shows a 3 cm extra-axial structure in the left frontal region, epidural in location.

**Figure 2:**
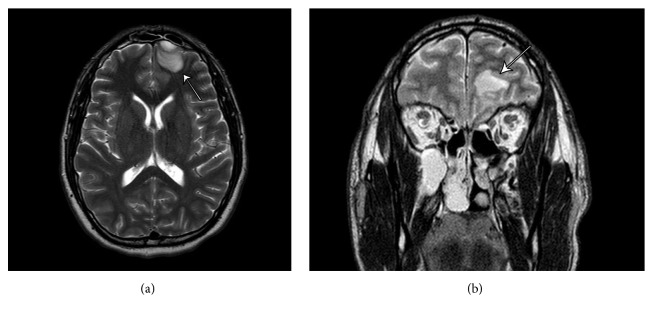
(a, b) show a 2.1 cm epidural lesion in the left frontal region producing minimal mass effect on the left frontal lobe with adjacent edema of the left frontal lobe and multichamber sinusitis (b).

Point-of-care glucose, complete blood count with differential, and basic metabolic panel were obtained, all within normal limits. Computed tomography (CT) of the head without contrast was ordered to evaluate for suspicion of intracranial pathology, revealing 1.3 cm attenuation behind the frontal bone and severe frontal sinusitis (Figure 1). At this point both neurosurgery and otolaryngology specialists were consulted. MRI with and without contrast were subsequently ordered, showing a 2.1 cm epidural lesion in the left frontal region producing minimal mass effect on the left frontal lobe (Figure 2).

While in the emergency department, the patient received Tylenol, empiric vancomycin, ceftriaxone, levetiracetam for primary seizure prevention, and dexamethasone to decrease intracranial edema. Over the next several hours the patient's fever decreased to 99°F and tachycardia resolved. However he still complained of frontal sinus pain and headache. The patient was admitted to the surgical intensive care unit (SICU) and scheduled for same-day craniotomy and sinusotomies. The patient was admitted to SICU for one week following the surgical intervention. His hospital course was uncomplicated. At the time of discharge the patient was asymptomatic, afebrile, and in stable condition. He was prescribed levetiracetam with follow-up after completion at neurosurgical, otolaryngology, and medicine clinics. Follow-up with the patient revealed two incidents of generalized seizures, likely due to postsurgical changes. Patient is kept on levetiracetam and doing well.

## 3. Discussion

This case emphasizes the importance of early recognition and treatment of intracranial suppurative complications of chronic sinusitis. The etiology of intracranial abscess encompasses a wide variety of pathologies. The most common include direct extension of osteomyelitis, mastoiditis, orbital cellulitis, otitis media, or direct/traumatic introduction of infection. Additionally common causes are iatrogenic, as consequences of intracranial procedures, and hematogenous seeding from a distant site of infection [3–5]. Our patient exhibited contiguous spread of infection from the frontal and ethmoid sinuses, which led to the formation of an intracranial abscess.

The clinical manifestations of an intracranial abscess are typically nonspecific, including headache (69%), fever (49–53%), seizures (25%), and nuchal rigidity (15%), resulting in delayed diagnosis; in the US the mean time to diagnosis is approximately eight days after the onset of symptoms [4]. Radiologic evaluation should be done in all patients suspected of intracranial complications of sinusitis. CT is often the first neuroimaging completed in the emergency setting and is considered the gold standard for properly visualizing the sinuses and intracranial structures; MRI is typically ordered prior to surgery due to higher resolution [4, 6]. It is also important to note that CT may not show fluid collection initially, so consideration must be given for repeat CT imaging or utilization of MRI. Opacification of sinuses with air-fluid levels and evidence of erosion of bone structures is possible in some cases [6].

It is important to reiterate that even though our initial suspicion of meningitis remained high lumbar puncture is contraindicated in patients with epidural abscess formation, especially if mass effect is appreciable on CT. Results are often nonspecific and neurologic decline as well as transtentorial herniation after lumbar puncture has been well documented and can lead to fatal consequences [4, 7, 8].

Anaerobic bacteria represent a common pathogen in the sequelae of complicated sinusitis presenting as intracranial abscess. Treatment of intracranial suppurative complication requires a combination of medical and surgical management. Medical management includes empiric antibiotic coverage with a third-generation cephalosporin and metronidazole [3, 9]. While aggressive broad-spectrum antibiotic treatment is universally accepted, it is important to realize that it is not an alternative to surgical management, and proper drainage of an epidural abscess should be carried out without delay [10, 11].

## 4. Conclusion

While intracranial complications are most commonly found in adolescent and young adult males, they have been reported at all ages. Our patient represents the typical sequelae in the course of complicated sinusitis. In this case we underscore the importance of early imaging in the presence of extrasinus manifestations. Furthermore, the literature illustrates that prompt management via craniotomy, sinusotomies, or both, in combination with medical management, proves to be most effective in preventing morbidity and mortality.
